# Characterization of factors influencing swarm dynamics and mating efficiency in *Anopheles coluzzii*

**DOI:** 10.1186/s13071-025-07151-w

**Published:** 2025-11-27

**Authors:** Tarwendpanga F. X. Ouédraogo, Simon P. Sawadogo, Abdoul Azize Millogo, Abdoulaye Niang, Judicael Ouedraogo, Seydou Bienvenu Ouattara, Antoine cribellier, Moussa Namountougou, Roch K. Dabiré, Florian T. Muijres, Abdoulaye Diabaté

**Affiliations:** 1 Institut de Recherche en Sciences de La Santé (IRSS), Direction Régionale de L’Ouest (DRO), 399 Avenue de La Liberté, 01 BP 545, Bobo-Dioulasso 01, Burkina Faso; 2 Université Nazi Boni, 01 BP 1091, Bobo-Dioulasso 01, Burkina Faso; 3 Institut des Sciences des Sociétés – INSS, 01 BP 7047, Ouagadougou 01, Burkina Faso; 4 Laboratoire d’Ecologie Vectorielle Et Parasitaire, Département de Biologie Animale, Université Cheikh Anta Diop, Dakar-Fann, Dakar, BP 5005 Sénégal; 5 Laboratoire d’Entomologie Fondamentale Et Appliquée (LEFA), Université Joseph KI-ZERBO, 03 BP 7021, Ouagadougou, Burkina Faso; 6 Experimental Zoology Group, Wageningen University & Research, Wageningen, The Netherlands

**Keywords:** Swarm, *Anopheles coluzzii*, Mating, Spatial distribution, Environmental factors

## Abstract

**Background:**

Malaria vectors reproduce through in-flight copulation within swarms, which remains poorly understood. Gaining insights into swarming and mating behavior is essential for optimizing novel vector control strategies including sterile insect technique, genetically modified mosquitoes, and behavior based intervention. This study investigates the factors influencing swarm dynamics and mating efficiency in *Anopheles coluzzii*.

**Methods:**

We surveyed swarms across 40 residential compounds in Burkina Faso, georeferencing swarming sites and recording swarming times and height. In a subset of three compounds selected for detailed characterization, we also measured inter-swarm distances, counted swarm size from photography, and mating pairs through direct observation during swarming. Furthermore, we collected 30% of male mosquitoes from swarms to measure wing length and perform PCR analyses. We monitored environmental variables including temperature, humidity, wind speed, and luminosity. Finally, we performed spatial and statistical analysis using ArcGIS and R to determine how swarm and mating dynamics are correlated and how they depend on biological and environmental conditions.

**Results:**

We identified 169 *Anopheles coluzzii* swarms and found strong evidence of spatial clustering (General G: *P* < 0.001; Moran’s *I* = 0.2, *P* < 0.001), with localized hotspots. Swarming occurred between 18:05 h and 18:45 h, extending into darkness at ~19:15 h. Swarms had an average height of 2.87 m (range: 1.0–3.2 m) and consisted of 83–2783 mosquitoes. Swarm size strongly predicted pairing success in *Anopheles coluzzii* (*t* = 9.16, *P* < 0.001) with larger swarms producing more pairs. However, individual pairing efficiency decreased with swarm size (*t* = −3.515, *P* < 0.001). Male size positively influenced individual pairing efficiency (*t* = 3.25, *P* = 0.002) but did not affect swarm size or total pairing frequency. Inter-swarm distances varied nonrandomly, suggesting interactions between neighboring and/or swarm markers.

**Conclusions:**

This study shows that *An. coluzzii* swarming is shaped by both biological and environmental factors. While larger males achieved higher individual mating efficiency, swarm size was the strongest predictor of mating success. Larger swarms yielded more mating pairs overall, although efficiency declined with increasing density. In addition, swarms formed in clustered nonrandom patterns within compounds. These results highlight the interplay between male traits and environment in shaping swarming dynamics.

**Graphical Abstract:**

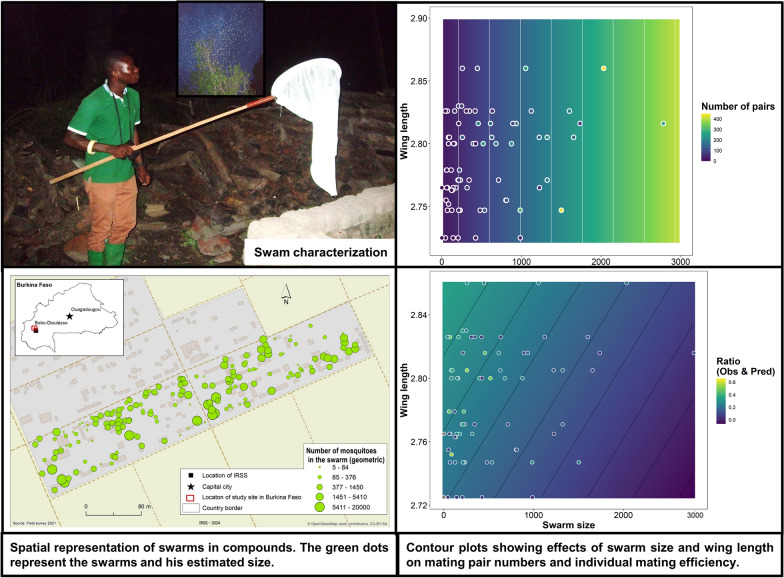

**Supplementary Information:**

The online version contains supplementary material available at 10.1186/s13071-025-07151-w.

## Background

Vector-borne diseases significantly burden global public health, particularly in tropical and subtropical regions [1, 2]. Malaria, a major vector-borne disease transmitted by infected *Anopheles* female mosquitoes, remains a major cause of death in Africa [3, 4]. In Burkina Faso, malaria remains endemic with peak transmission occurring from June to October [5–7].

Traditional vector control strategies, such as indoor residual spraying and long-lasting insecticidal nets, have contributed to reducing malaria incidence, but their effectiveness is increasingly undermined by insecticide resistance and changes in mosquito behavior, including increased outdoor biting at dawn and dusk [8–11]. Considering that new insecticide formulations may face resistance owing to natural selection [12], it is crucial to explore complementary and innovative tools to sustain progress towards malaria elimination.

Understanding mosquito ecology, especially reproductive behavior, is essential for developing effective interventions [13]. While previous research has focused primarily on female mosquitoes that transmit parasites to humans, new control strategies are emerging on the basis of an understanding of mosquito mating biology [13–15]. Among available methods, the sterile insect technique [16, 17] and genetically modified mosquitoes are more promising [18, 19]. However, the effectiveness of these methods depends on the modified mosquito’s ability to mate with wild populations. Thus, a deeper understanding of mosquito reproductive ecology, including male biology and mating behavior is essential [16, 17]. In *Anopheles coluzzii*, mating occurs in male-dominated swarms at specific landmarks, but key aspects of swarm behavior and organization remain poorly understood under natural conditions [18–20]. These swarms are typically ephemeral, forming 10 to 30 min after sunset depending on the species, and are spatially scattered across the landscape [21]. They often comprise hundreds to thousands of males, while females join only briefly to mate before quickly departing [22, 23]. Furthermore, studies have shown that swarming is influenced by environmental cues, male density, and competition and that swarming sites are often stable over time [20, 24, 25]. Despite recent advances, key aspects of swarming behavior such as the determinants of swarm size, the influence of individual phenotypic traits, and the mechanisms of female mate choice remain poorly understood in natural conditions. The ephemeral and dispersed nature of mosquito swarms further complicates their detection and targeting for vector control [26] although specialized imaging and tracking systems have been used in other work to capture swarm dynamics at high resolution [25, 27–31]. Addressing these gaps is crucial for the development of innovative and effective control strategies.

Thus, we conducted a detailed quantitative study of *Anopheles coluzzii* swarms in the Vallée du Kou, characterizing key parameters involved in swarm formation and spatial distribution. By elucidating the factors that govern swarm structure and location, our work aims to provide essential insights for designing novel interventions targeting this critical stage in the mosquito life cycle.

## Methods

### Study area

**Fig. 1:**
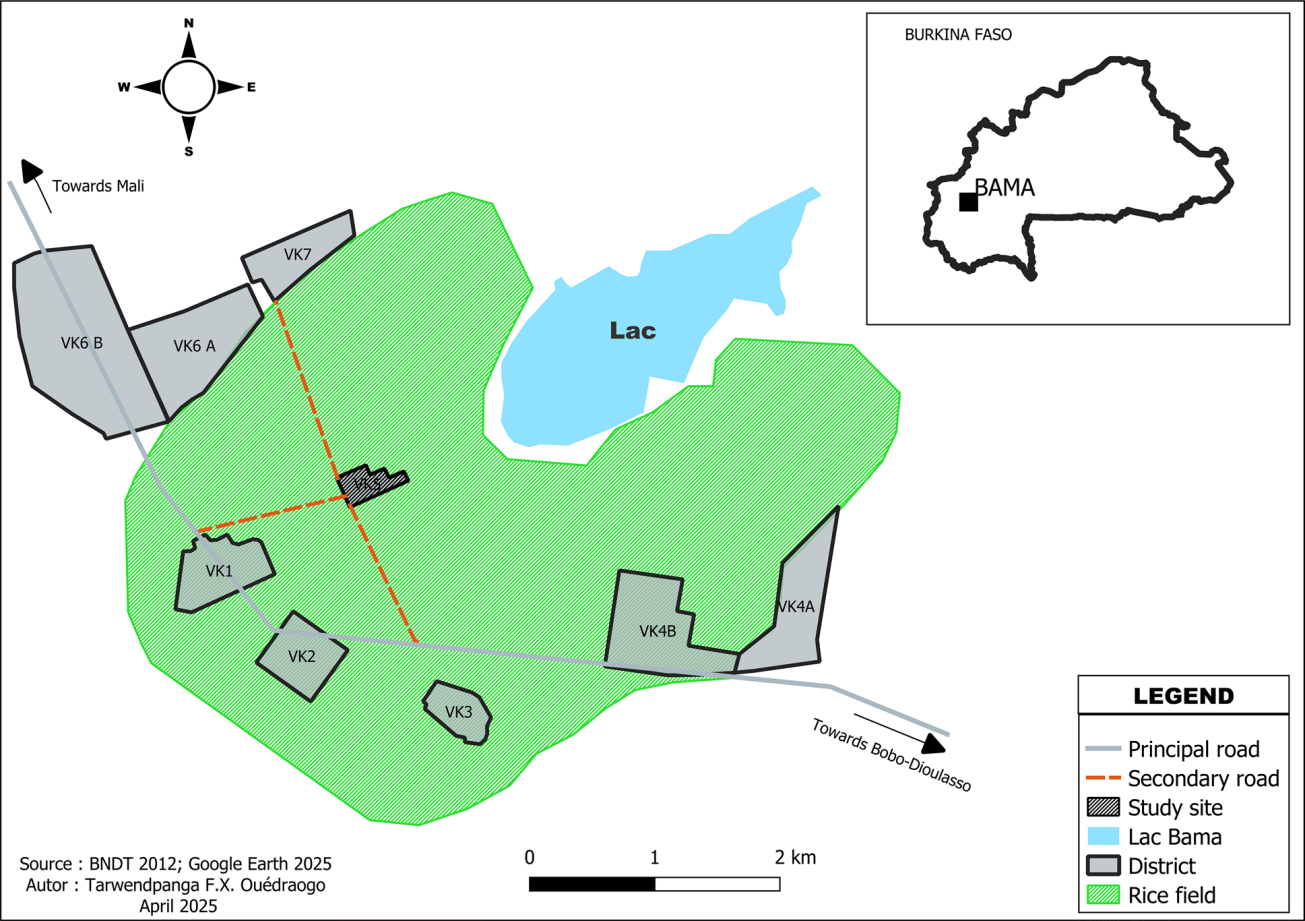
Location of study site

The study was conducted in 40 residential compounds (household units consisting of one or more inhabited houses) in VK5, a district of Bama village (Fig. 1). Bama is located 30 km northwest of Bobo-Dioulasso in the Western Burkina Faso (4° 24′ W; 11° 24′ N), characterized by rice fields. VK5 is characterized by its surrounding rice paddies that serve as permanent breeding grounds for mosquito larvae. The irrigation system within the rice fields creates persistent breeding sites for larvae, facilitating the continuous proliferation of *An. coluzzii* throughout the year [32]. During the 1970s, as part of an irrigation development initiative, seven villages were established over an area of 7200 hectares. This region experiences two distinct seasons: the rainy season, from May to October, and the dry season, lasting from November to April [21]. Malaria transmission in Bama is primarily driven by *An. gambiae* s.l., with *An. funestus* playing a secondary role [33].

### Swarm characterization

Swarm identification was undertaken by trained local volunteers under the supervision of the field entomology technicians. Once located, swarms were georeferenced using a GARMIN GPSMAP^®^ 62, with latitude and longitude coordinates recorded to an accuracy of ± 2 m. In all 40 residential compounds, we estimated swarm height from the ground to the bottom of the swarm using a graduated stick. During each observation session, environmental parameters including temperature, relative humidity, wind speed, and luminosity were recorded using an MSR mini data logger. Owing to logistical constraints, it was not feasible to monitor all swarms simultaneously across the 40 compounds. Therefore, three compounds were selected for detailed monitoring based on criteria such as geographic distribution, swarm size and accessibility. Regarding the geographic distribution, we selected houses located in different areas to cover a diversity of locations within the neighborhood. In addition, we then also considered the previously estimated swarm sizes to choose houses that exhibited variation in swarm size. We also considered the accessibility of the houses to facilitate measurements and capture swarm’s photos. Finally, we selected houses that were representative of the overall identified swarms with a presence of at least five permanent swarms. We performed a selection from the available compounds to ensure diversity and representativeness in the chosen sample. Based on these selection criteria, compounds 6, 15, and 21 were selected for detailed observation and measurement of parameters. Thus, we sampled mosquitoes within swarms using aerial sweep nets. Species identification was conducted on 30% of the collected specimens from each swarm, following the molecular protocol described by Santolamazza et al. [34].

### Collection of resting mosquitoes

Resting mosquito populations were sampled through indoor spraying using a nonresidual insecticide. Spraying was conducted from 7:00 AM to 9:00 AM in the same compounds monitored during the swarming study period. This sampling method gave an accurate estimate of the total density of mosquito species in our study site [35].

### Measurement of inter-swarm distances

The spatial distribution of swarms within each compound was mapped using GPS coordinates recorded at individual swarm locations. These data were imported into ArcGIS software version 10.8 to calculate the Euclidean distances between all swarming points within the compound. This approach enabled a detailed analysis of the spatial arrangement and clustering patterns of *Anopheles* swarms, yielding important insights into dispersal behavior and potential site fidelity factors critical for developing swarm-targeted control strategies.

### Determining swarm size and mating pair frequency

**Fig. 2:**
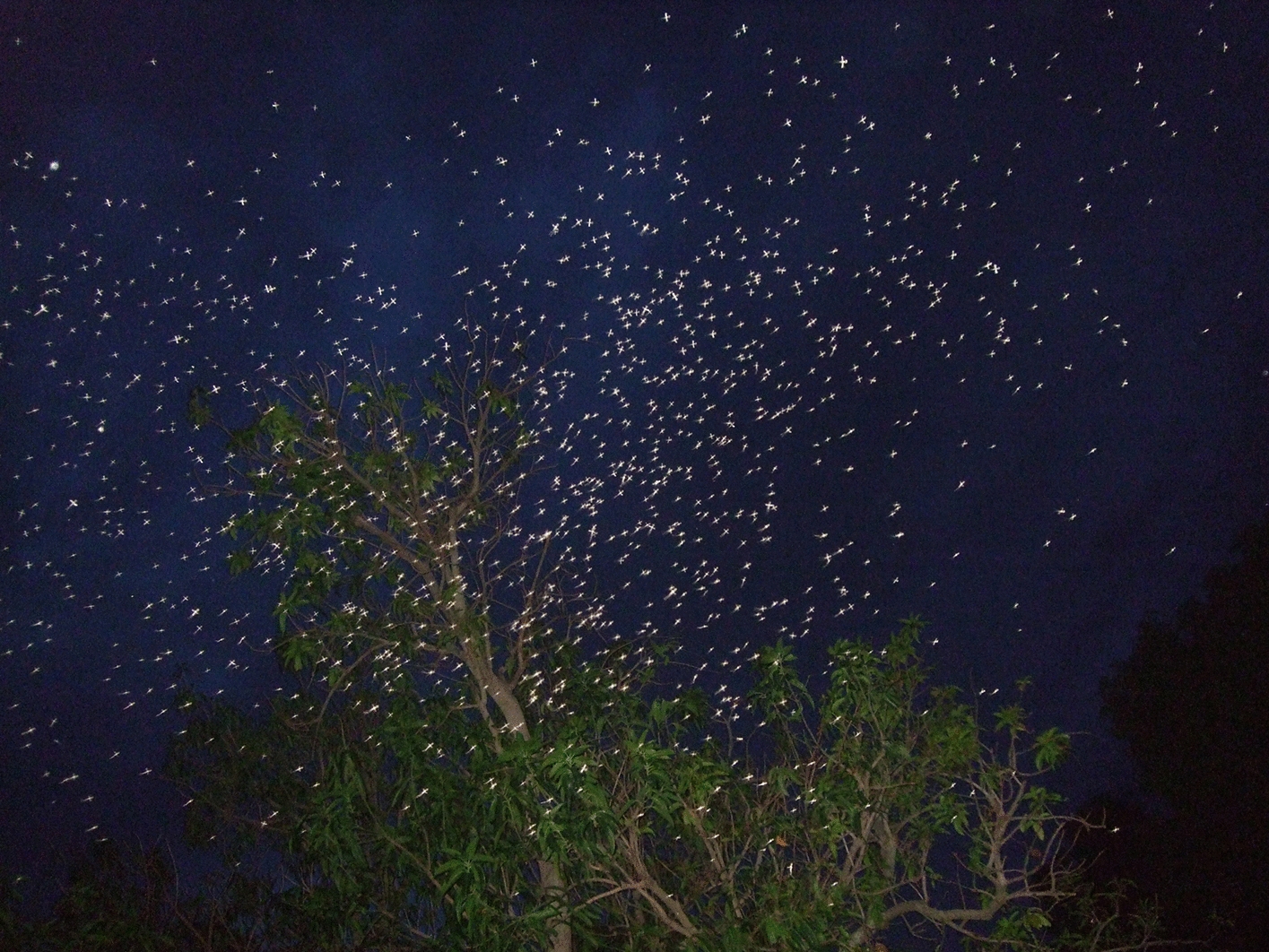
*Anopheles coluzzii* swarm in VK5. White dots in aggregations are mosquitoes (Photo: Ouédraogo 2021)

At each identified swarming site, two volunteers were deployed during the swarming period. A Fujifilm F40fd 8.3-megapixel camera was used by the first to capture images of the swarm every 5 min between 6:00 PM and 7:20 PM. These photographs enabled precise counting of mosquitoes within the swarm (Fig. 2). The second one used a tally counter to record the number of mating pairs from the swarm during the same time points, recognizing that these observations may underestimate actual copulation frequency. Images of swarms were later transferred to a computer and analyzed using ImageJ software (1.45 Wayne Rasband, NIH, USA) to determine swarm size by counting individual mosquitoes. Mosquito counts were performed in ImageJ using a sequential marking technique that automatically assigned a number to each mosquito as it was selected. To ensure accuracy, every picture was counted three times, and the results were cross-checked to confirm the exact number of mosquitoes.

### Mosquito collection from the swarms

Mosquitoes were collected from the swarms 10 min after their appearance using sweep nets [36]. To standardize sampling, a single sweep was performed per swarm. These collections were conducted in all three selected compounds. Subsequently, the collected mosquitoes underwent identification under a binocular microscope using morphological identification keys [37]. Male mosquitoes were sorted and preserved in groups of five in 1.5 ml micro-tubes filled with 80% ethanol for subsequent morphometric analysis.

### Wing dissection and measurement

Mosquito wing length is considered an indicator of their body size [38]. Thus, preserved male mosquitoes were collected in each swarm, dissected mounted dry on microscope slides, and then photographed using a Leica EZ4 D microscope (Leica Microsystems, Switzerland) connected to a computer. The length from alula notch to the wing tip [39] were measured using Image J software (1.45.0 Wayne Rasband NIH, USA).

### Data for 3D visualization

In addition to GPS coordinates, sketches of the three compounds were drawn on paper sheets for 3D visualization of the swarm’s position. The maps represented physical components of the three selected compounds such as walls, buildings, trees, wells and toilets, wood piles, clay pots, with information on their length, width and height, color, and relative shape of each. The field sheet was used to digitize the feature in Arc Maps 10.8. The digitization was assisted by online ArcGIS images accessible in Arc Map. The results of the digitization were imported into Arc Scene for 3D visualization and interpretation. Variations in the green colors of the tree have been inserted to improve visualization.

### Data analysis

We used ArcMap 10.8 (ArcGIS, ESRI, USA) for two-dimensional mapping and spatial analysis, and ArcScene for three-dimensional visualization. The spatial structure and clustering of swarm distributions were assessed using the Spatial Autocorrelation Getis-Ord General G statistic and Moran’s I. Inter-swarm distances were calculated using the Euclidean distance tool. All graphical representations and statistical analyses were performed with R software version 4.3.2. We performed principal component analysis (PCA) to reduce the dimensionality of dataset and to visualize the major patterns of variation among key variables. Differences in wing length across swarms were assessed using analysis of variance (ANOVA). The effects of wing length and swarm size on pairing success were analyzed using general linear mixed models (GLMMs), retaining 95% confidence intervals for all variables included in the final minimal adequate model. Swarm heights are reported as mean ± standard error (SE). Key swarm characteristics including average height above ground, mean swarm size, and swarm duration were also quantified.

## Results

### Temporal dynamics of swarming activity

Swarming activity was observed to begin from 6:05 PM to 6:45 PM, with dispersal occurring from 6:35 PM to 7:15 PM, resulting in an average swarm duration of approximately 27 min. Swarms typically formed at an average height of 2.87 ± 0.20 m above ground level.

### Spatial distribution of swarming sites and clustering analysis

**Fig. 3:**
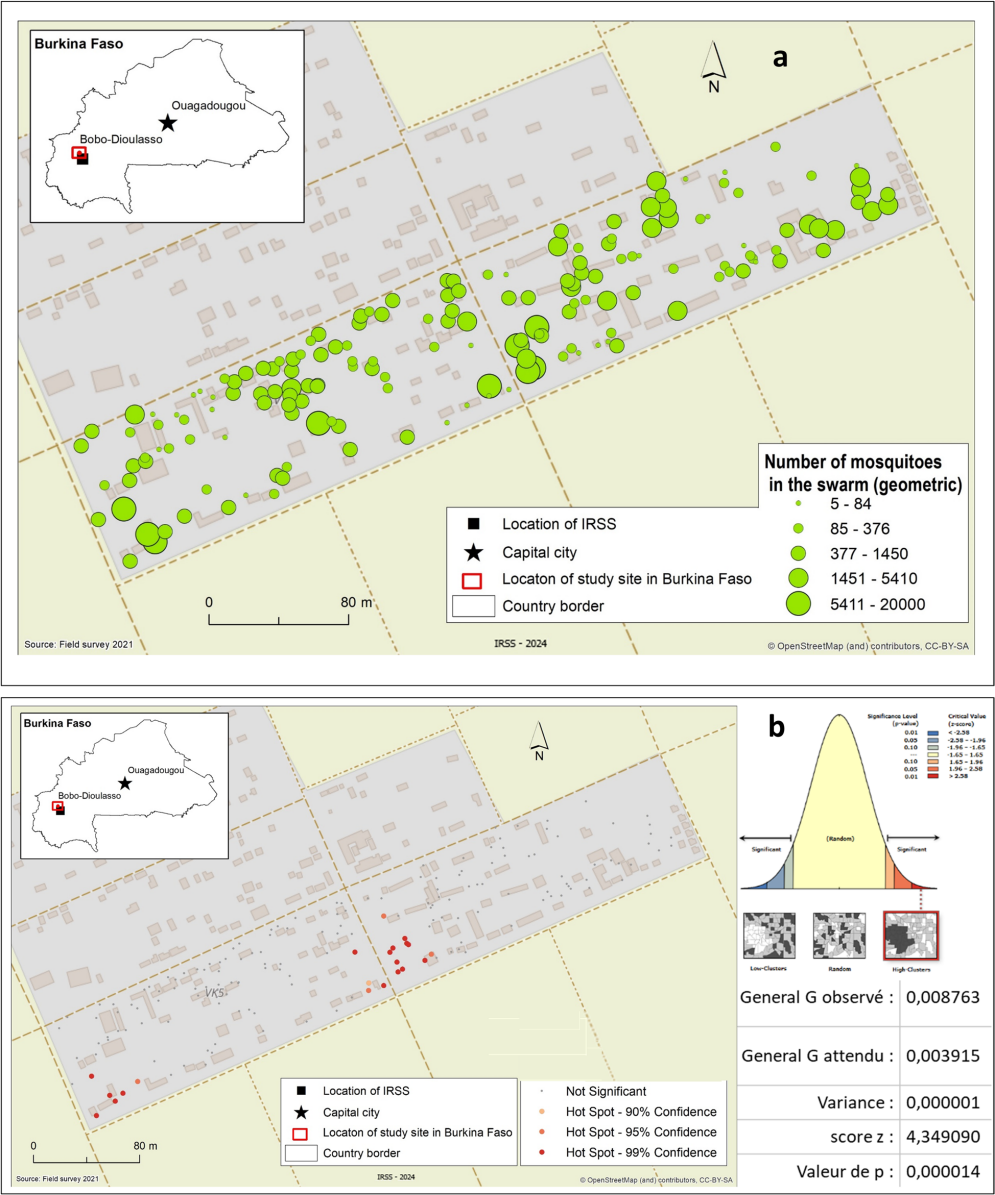
Spatial representation and clustering of swarms in compounds. The green dots represent the swarms and his estimated size **a**. Geometric discretization has been applied to mitigate the high variability in the swarm sizes. Significant hot spots (red) were identified using the Getis–Ord General G statistic (Observed *G* = 0.008763, *P* < 0.001). Hot spots indicate high-value clustering and nonsignificant areas are grey **b**. The inset map shows the study site within Burkina Faso. The significance plot and summary (*Z* = 4.35, *P* = 0.00014) indicate a strong deviation from randomness, confirming pronounced clustering of hot spots in the study area

Surveys across 40 compounds in VK5 identified 169 swarms that were clustered near human dwellings. The number of swarms per compound ranged from 3 to 12. Overall, results indicated a mean nearest-neighbor distance shorter than expected under spatial randomness and in some areas the swarms had a slightly higher density than other areas (Moran’s Index = 0.2; *P* < 0.001). Figure 3 shows the spatial distribution of swarms as a function of the number of *Anopheles coluzzii* males (Fig. 3a). The spatial autocorrelation and general G statistics reveal a clustered, nonrandom swarm distribution with distinct hot spots (Fig. 3b). Visual evidence of clustering is statistically confirmed by a General G-statistic (observed = 0.008763 versus expected = 0.003915; *Z* = 4.349090, *P* < 0.000014), indicating that the observed clustering is unlikely to result from random processes. The inset shows statistically significant hot spots at 90%, 95%, and 99% confidence levels, highlighting areas of intense swarm activity and potential vector control interventions. These results provide evidence of nonrandom swarm organization. The observation of few nonsignificant swarming locations suggests a random spatial distribution of both large and small swarms.

### Spatial distribution of swarm and mating pairs

**Fig. 4:**
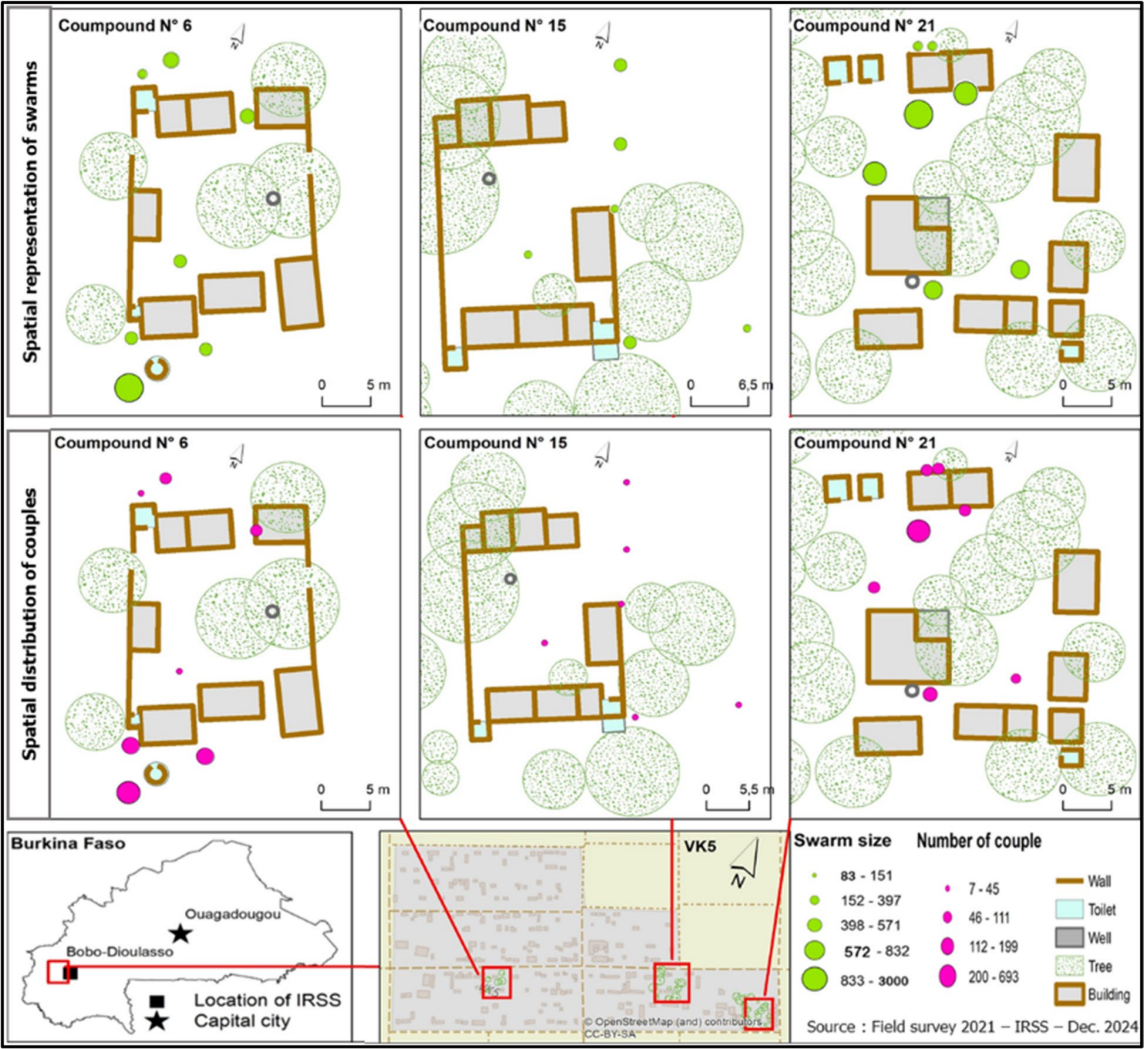
Spatial distribution of swarms and mating pair in the three compounds. The figure illustrates the spatial arrangement of swarms (top row) and mating pairs (bottom row) in compounds 6, 15, and 21. Green circles represent swarm size, while magenta circles indicate the number of mating pairs. Swarms cluster near buildings and vegetation, with compound 21 showing the largest swarms and highest pairing activity. The inset map shows the study location in Burkina Faso and the layout of the compounds, while the legend links swarm size and mating pair counts, highlighting a positive association between these variables

Figure 4 illustrates the spatial distribution and characteristics of *Anopheles coluzzii* swarms and mating pairs across three monitored compounds. All swarms were observed in open areas devoid of overhead obstacles. Swarm sizes as determined by photographic captures ranged from 83 to 2783 mosquitoes while the number of mating pair per swarm ranged from 7 to 693. Larger swarms exhibited significantly higher number of mating pairs consistent with previous findings on density-dependent mating success. Spatial heterogeneity was observed with compounds 6 and 21 showing higher densities of both swarms and mating pairs. However, variation in the average number of mating pairs per swarm varied across compounds suggests that factors beyond swarm size such as microhabitat characteristics or male quality may influence mating success (Fig. 4).

### 3D visualization of swarms

The 3D visualization shows that swarms typically form below the tree canopy, favoring partially sheltered areas that offer wind protection while maintaining open horizons for dusk luminosity (Supplementary Fig. S1). This approach provides a detailed understanding of swarm positioning within the landscape, highlighting the influence of tree structure on swarm formation. Overall, the visualization reveals the strong interaction between swarm dynamics and environmental features, offering precise insights into how natural habitat characteristics shape mating swarm behavior.

### Principal component analysis (PCA) of swarming parameters

**Fig. 5:**
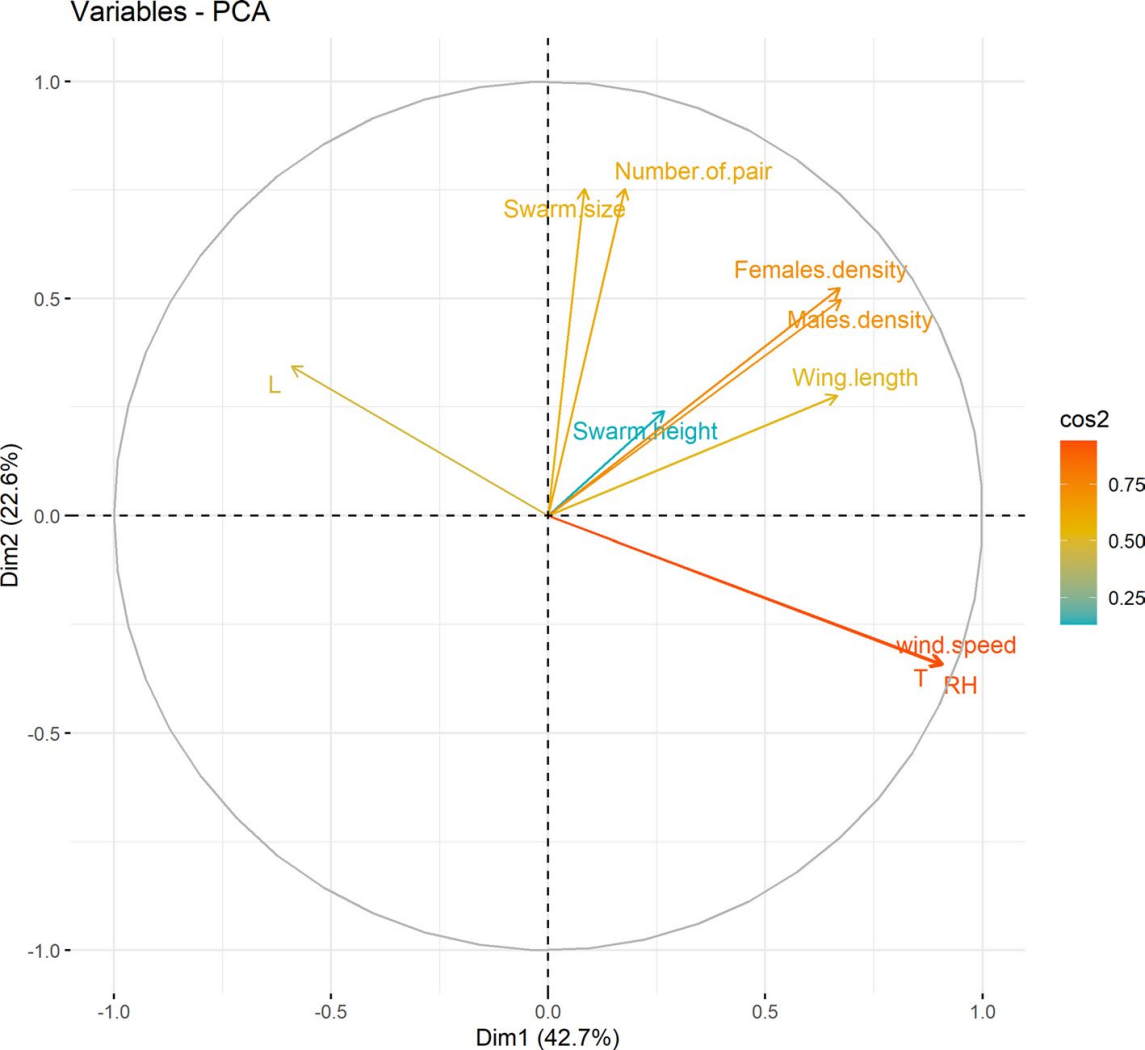
Principal component analysis (PCA) of swarming parameters, T = temperature, L = luminosity, RH = relative humidity

The PCA biplot (Fig. 5) shows the multivariate relationships between biological and environmental factors affecting *An. coluzzii* swarms. The first two components explain 65.3% of total variance. Dim1 is strongly driven by biological traits (swarm size, mating pairs, wing length, male, and female densities), while Dim2 reflects environmental variation. Luminosity (L) is negatively correlated with biological parameters, indicating reduced swarm activity under low light. Swarm height, wind speed, temperature (T), and relative humidity (RH) exhibit weaker associations with both components. Overall, PCA suggests that swarming behavior is primarily shaped by biological factors, with environmental variables acting as secondary.

### Interaction between male body size and swarming sites

**Fig. 6:**
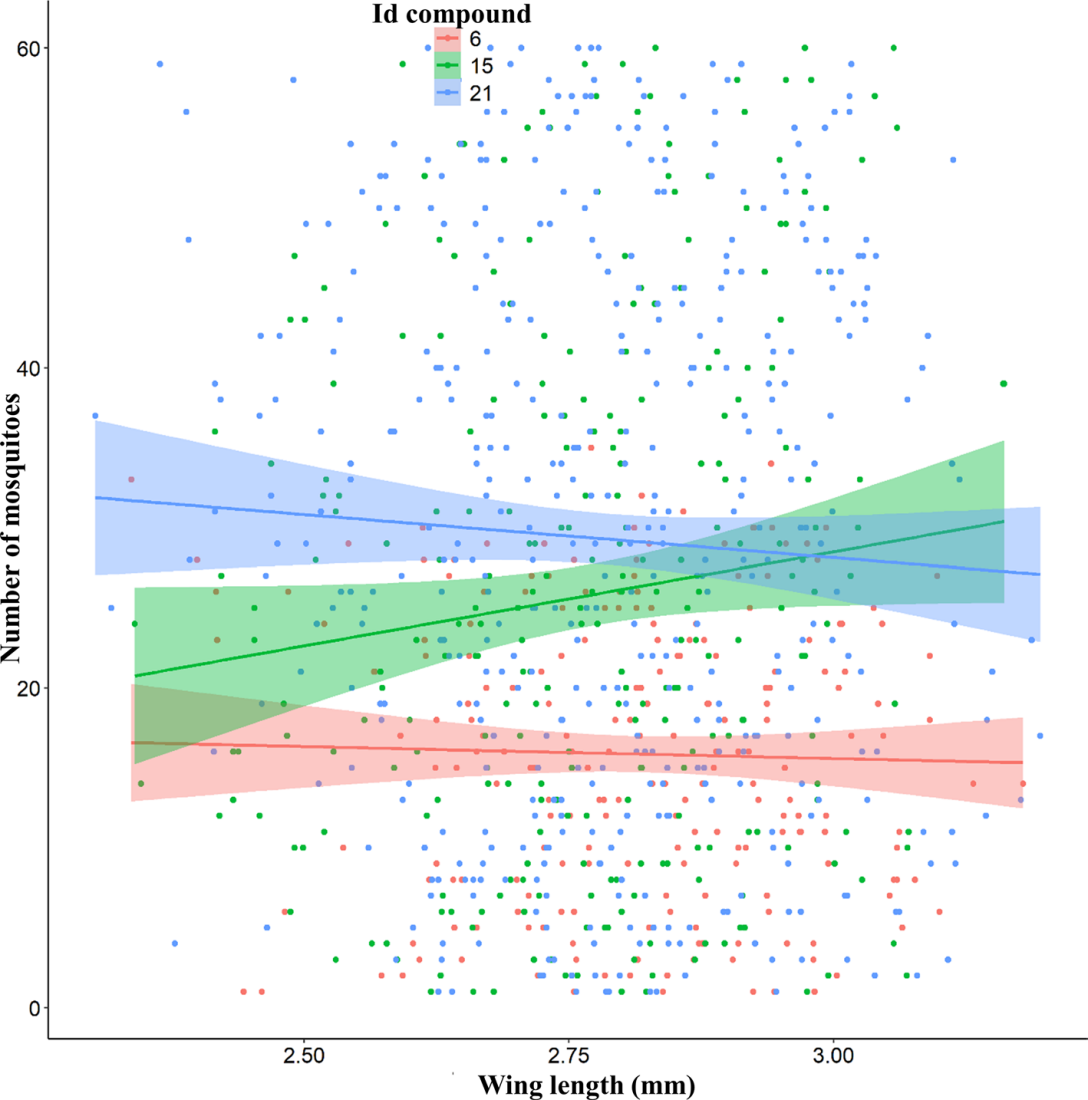
Relationship between male body size per compound. Each point represents the number of mosquitoes whose wings were measured in the compound ± standard error (es) as a function of wing size. The pink, green, and blue lines represent the respective linear regression lines

A total of 1000 male mosquitoes from swarms at the three compounds were dissected to measure their wing length. Wing length was used as a proxy for male body size. Wing length varied among compounds with a mean of 2.82 ± 0.18 mm in compound 6, 2.77 ± 0.15 mm in compound 15, and 2.78 ± 0.13 mm in compound 21. No significant relationship was found between male body size and number of mosquitoes in the swarm (*t* = 1.868, *p* = 0.06898; Fig. 6).

### Relationship between swarm size, male size, and mating pair formation

**Fig. 7:**
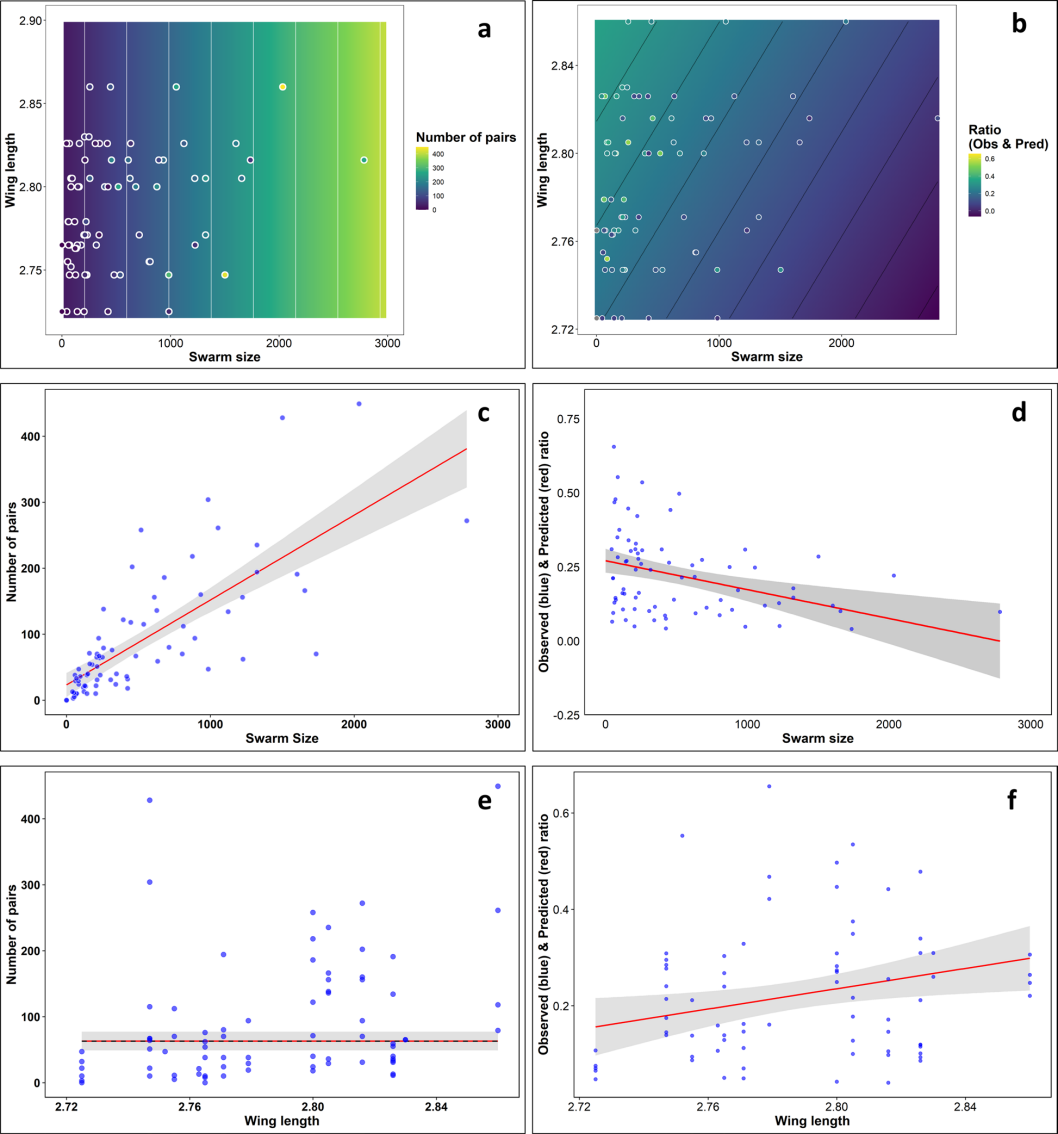
Effect of swarm size and male wing length on (**a**–**c**) the number of mating pairs, and (**d**–**f**) the individual mating pair efficiency. Panels (**a**) and (**b**) present contour plots showing the combined effects of swarm size and wing length, with panel (**a**) highlighting the predicted number of mating pairs and panel (**b**) illustrating individual mating efficiency (ratio of observed to predicted pairs per male). Panel (**c**) shows a strong positive relationship between swarm size and the total number of mating pairs, while panel (**d**) reveals a negative association between swarm size and individual mating efficiency, indicating reduced pairing success per male in larger swarms. Panels (**e**) and (**f**) display the effects of wing length, with a weak, nonsignificant influence on total pairs (**e**) but a slight positive trend on individual mating efficiency (f). Points show sampled data, and shaded trend lines show the estimates and 95% confidence intervals from the generalized linear mixed models (GLMMs), for the mean swarm or wing size, respectively. Solid and dashed lines show significant and nonsignificant trends, respectively

The generalized linear mixed model (GLMM) analyses demonstrated that swarm size is the strongest predictor of mating success in *Anopheles coluzzii*. As illustrated in Fig. 7a, c, the total number of mating pairs increased significantly with swarm size (*t* = 9.16, *P* < 0.001), establishing swarm size as the principal driver of pairing. However, individual mating efficiency (ratio of pairs per swarming male) declined as swarm size increased (Fig. 7b, d), indicating density-dependent constraints that reduce pairing success under high male densities. Male wing length, used as a proxy for body size, had no significant influence on the total number of mating pairs (*t* = 1.49, *P* = 0.14; Fig. 7e), but showed a slight positive association with individual mating efficiency (Fig. 7f), suggesting that larger males gain a marginal advantage under low-density conditions. The contour plots (Fig. 7a–b) further highlight the joint effects of swarm size and body size: while swarm size dominates total pair formation, pairing efficiency is highest in small, less dense swarms with larger males and lowest in large swarms with smaller males. Overall, these results underscore that collective swarm dynamics overshadow individual morphological traits, with large swarms generating more mating pairs but reducing individual success rates due to density-dependent competition.

### Influence of distance between swarming sites

**Fig. 8:**
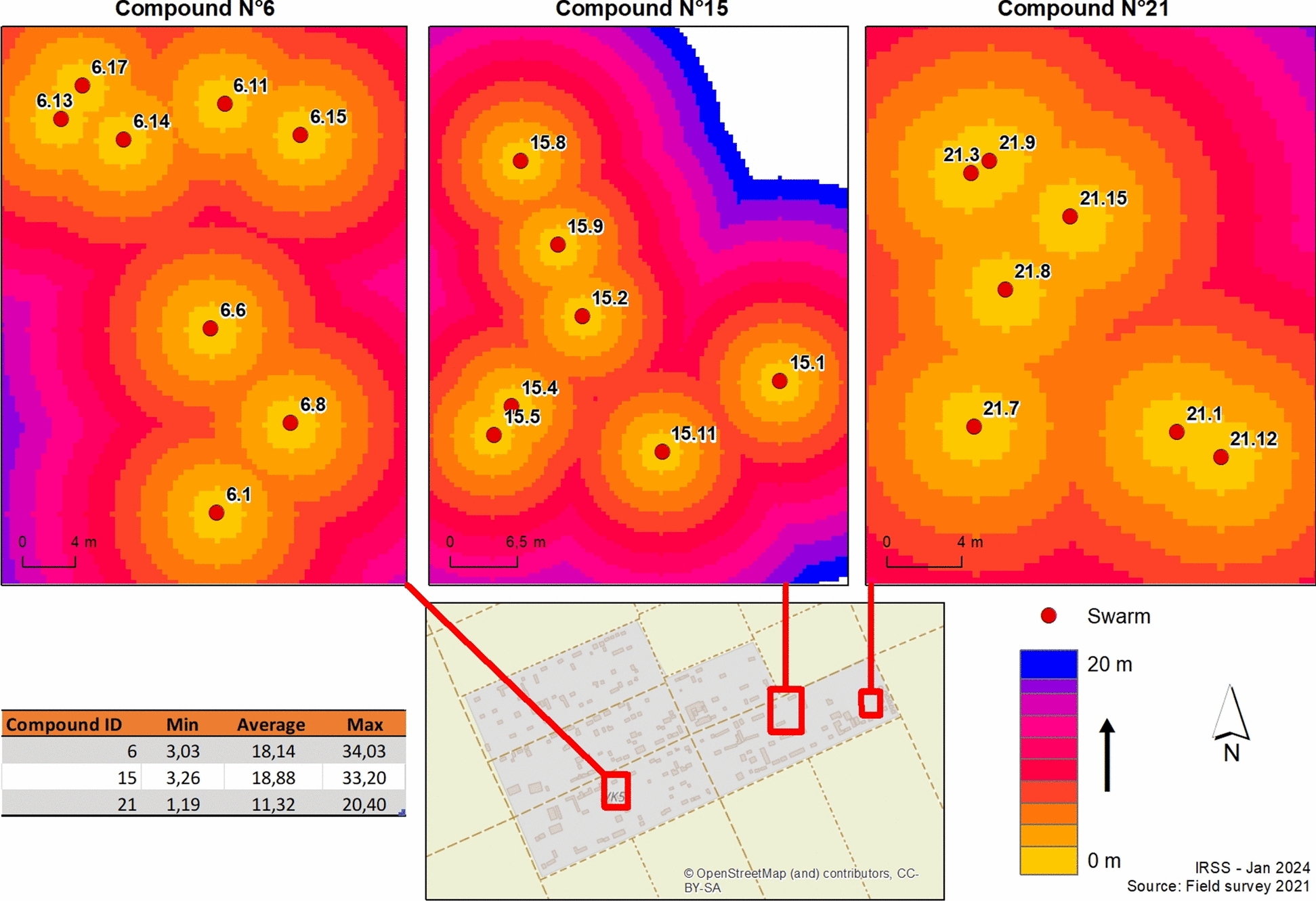
Spatial representation of swarm distance. Each number represents a swarm id and red dots represent swarms. The different color shades represent the distances between swarms

The spatial distribution of swarming sites in compounds 6, 15, and 21 showed notable variation in inter-swarm distances, with patterns suggesting localized clustering. Swarms’ spatial structure within each compound is presented in Fig. 8, complemented by tables reporting the minimum, mean, and maximum inter-swarm distances across the study area. Compound 21 demonstrated the most tightly clustered swarm distribution with an average inter-swarm distance of 11.32 m. In contrast, compounds 6 and 15 exhibited greater mean distances of 18.14 m and 18.88 m, respectively, along with increased variability in spacing between swarms. This spatial analysis suggests potential variations in environmental factors or compound-related influences on swarm location and density, necessitating further investigation into their respective roles in local *An. coluzzii* ecology.

## Discussion

The temporal pattern of swarming behavior in *An. coluzzii*, beginning from 6:05 PM to 6:45 PM and dispersing after 7:15 PM, aligns with prior studies. The variation in start and end times across months indicates possible seasonal effects on swarming behavior, necessitating further investigation into the underlying climatic drivers [21, 40]. In addition, the use of flash cameras in this study allowed the detection of swarm activity even after sunset.

Beyond temporal dynamics, environmental factors played a central role in shaping swarm distribution. A total of 169 swarms were observed across 40 residential compounds, with the number of swarms per compound ranging from 3 to 12. This high density of swarms is likely influenced by environmental factors such as the consistent water availability during the rainy season and proximity to rice fields, both of which are known to provide ideal larval breeding sites for *Anopheles coluzzii* [20, 41, 42] and the presence of swarm markers. Similar observations have been reported in other studies where wood bundles, rubbish piles, and wells serve as swarm markers [22].

The clustering of swarms around human residences aligns with previous studies that have highlighted the attraction of mosquito swarms to human activity areas [21]. Moran’s index of 0.2 and a highly significant *P*-value confirm a nonrandom distribution, reinforcing the role of environmental and anthropogenic factors in swarm formation and identifying hotspots that could be prioritized in vector control strategies. The spatial configuration of swarms within compounds exhibited clustering, likely influenced by environmental obstacles such as tree branches and foliage, which necessitate swarming in open spaces. While the exact swarming distance was not consistent, the clustering of swarms suggests that specific environmental cues or spatial needs drive swarm formation in particular areas. This observation is consistent with studies showing that mosquitoes avoid obstacles detected by changes in airflow [43]. While further investigations are needed to identify intrinsic factors that influence swarm positioning, it can be suggested that the positioning is related to the presence of specific markers. Swarms tend to form above specific markers, sometimes near each other. These findings are consistent with the hypothesis that swarm positioning is guided by the availability of specific visual markers, which may cluster spatially and influence swarming.

The observed mean swarm height of 2.87 m likely reflects an adaptive preference for open spaces at sunset, facilitating aggregation and visibility to females while avoiding obstructions such as house walls and tree foliage. Environmental features at sites such as VK5, where obstacles are present, may drive swarms to form at higher elevations to maintain clear lines of sight to the western horizon, consistent with previous findings that obstacles influence swarm height variability [44].

The intrinsic biological parameters were the primary measured drivers of variation, although unmeasured micro-environmental variation may also play an important role. The negative correlation between luminosity and biological parameters suggests reduced swarm activity under low light conditions. Environmental cues and behavioral components remain complex as reported [13]. These interactions highlight the need for future modeling efforts that integrate both biological and environmental predictors.

Our analyses provide clear evidence that swarm size is the strongest measured predictor of pairing success in *Anopheles coluzzii* mosquitoes. As shown in results, the number of mating pairs increases with swarm size, indicating that bigger aggregations offer more opportunities for pair formation, confirming that larger swarms are associated with increased pairings [17, 45]. This finding aligns with previous studies, which demonstrate that bigger swarms are more easily detected by females owing to their greater size, collective wingbeat sounds, and potentially higher pheromone production [13, 46]. However, our results also show that mating efficiency per individual declines as swarm size increases, indicating density-dependent constraints or heightened competition among males. Notably, while the number of pairs rises with swarm size up to an optimal threshold of approximately 1000 mosquitoes [47], observed mating frequency may decrease or be underestimated in large swarms. This is likely owing to observational bias, as the constant movement and dispersal of pairs in all directions make accurate visual counting challenging. In addition, occasional imbalances in the male-to-female ratio may further contribute to reduced observed mating in large swarms. These findings highlight that while larger swarms promote more pairing events, other factors must be considered when interpreting mating success, particularly in large swarms.

Body size analysis revealed that the swarms consisted of males with a wide range of body sizes, with no significant bias toward larger or smaller individuals, consistent with a previous study [21]. This suggests that the swarming behavior of *An. coluzzii* in VK5 is independent of mosquito size. However, our results also show a modest, though not statistically significant, positive association between male body size and the number of mating pairs, as well as a slight increase in pairing efficiency with bigger body size. While these trends did not reach statistical significance in our field study, they are in line with previous reports that larger males may achieve higher mating success owing to competitive advantages such as improved flight performance, acoustic signaling, or pheromone production [16, 25, 30]. The importance of male body size in determining male mating competitiveness is further supported by laboratory studies showing that larger males are more likely to acquire a mate than smaller males in *An. gambiae* [16, 21, 25].

## Conclusions

This study revealed that *An. coluzzii* swarms are distributed in a nonrandom, clustered pattern, with clearly defined hot spots that are closely associated with specific environmental markers. Swarming behavior lasted an average of 27 min and the use of flash photography allowed detection of mosquitoes in swarms beyond sunset, expanding our understanding of swarm persistence. Our results showed that swarm size is the strongest measured predictor of pairing success: larger swarms consistently produced more mating pairs, although individual mating efficiency declined with increasing swarm size, suggesting density-dependent constraints. By linking swarm characteristics with environmental and biological factors, we highlight key determinants of swarm accessibility and mating efficiency. Looking ahead, integrating broad-scale mapping with fine-scale behavioral monitoring will provide a more complete picture of swarm ecology and directly inform the development of targeted intervention strategies.

## Supplementary Information

Supplementary material 1. Fig. S1 3D visualization of swarms

## Data Availability

Data supporting the main conclusions of this study are included in the manuscript.

## Abbreviations

3D: Three-dimensional
sl: Sensu lato
spp: Multiple species of the genus
PCR: Polymerase chain reaction
VK5: Vallée du Kou district 5
GPS: Global positionning system
MSR: Measurement storage and recording
