# Supplementary figures and images for: Characterization of factors influencing swarm dynamics and mating efficiency in Anopheles coluzzii
Source: Parasit Vectors. 2025 Nov 27;18:512. doi: 10.1186/s13071-025-07151-w (PMC12751959; doi:10.1186/s13071-025-07151-w)

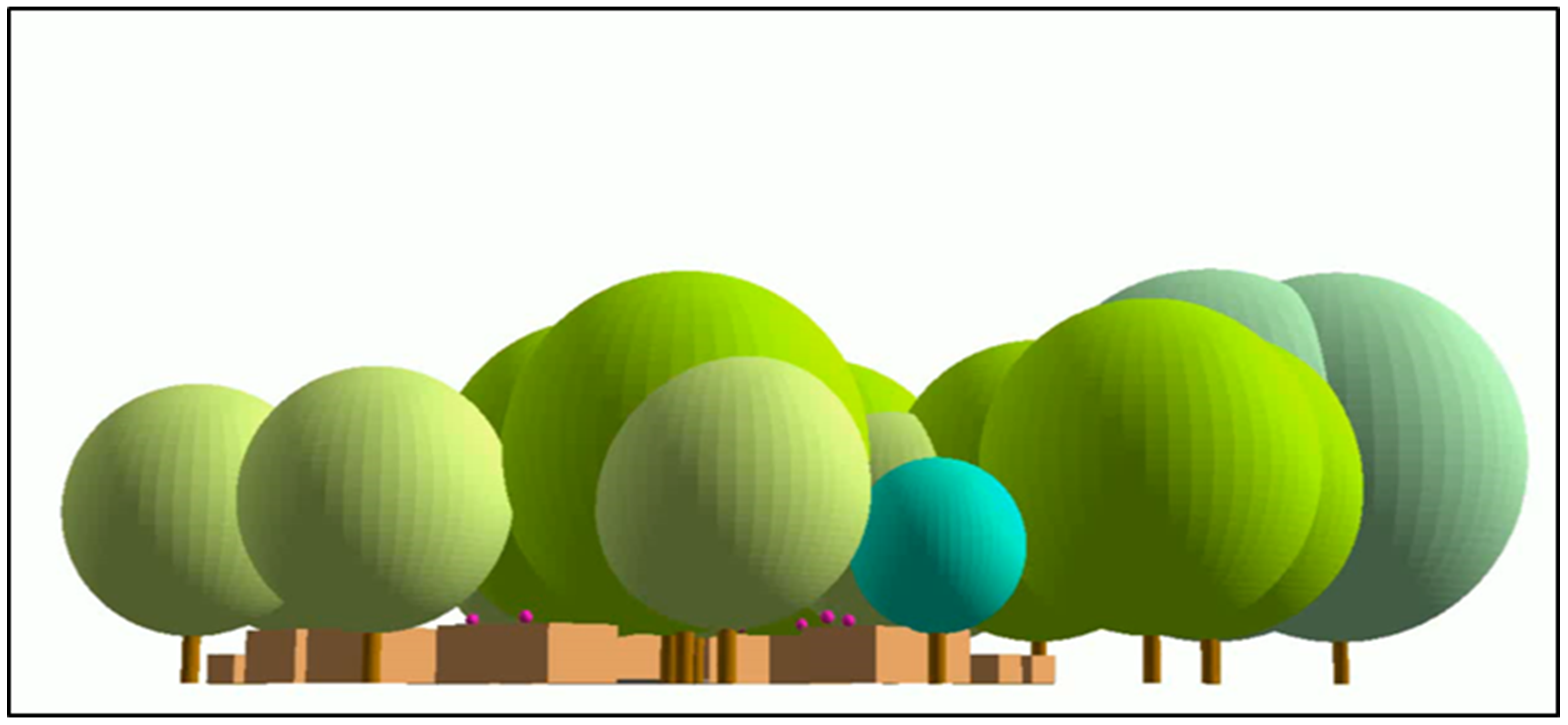

Supplement: Supplementary file 1 — Supplementary material 1. Fig. S1 3D visualization of swarms [file 13071_2025_7151_MOESM1_ESM.tif]
